# Acute ST-segment elevations following paclitaxel administration for uterine cervical cancer: a case report and literature review

**DOI:** 10.1186/s40959-022-00148-9

**Published:** 2022-12-01

**Authors:** Shota Higami, Yusuke Tanaka, Tomomi Deguchi, Mariko Shiraishi, Yasuhiko Shiki

**Affiliations:** 1 Department of Obstetrics and Gynecology, Osaka Rosai Hospital, 1179-3 Nagasone-Cho, Kita-Ku, Sakai, Osaka 591-8025 Japan; 2 Department of Obstetrics and Gynecology, University of Occupational and Environmental Health, Kitakyushu, Fukuoka Japan

**Keywords:** Cardiac ischemia, Chemotherapy, Gynecologic malignancy, Paclitaxel, Paclitaxel-induced myocardial infarction, ST elevation

## Abstract

Paclitaxel-induced cardiac ischemia is a rare but life-threatening complication. Although it may be difficult to distinguish from hypersensitivity or infusion reactions, it should not be overlooked. We herein report a rare case of ST-segment elevation following the administration of paclitaxel for uterine cervical cancer and review the literature regarding paclitaxel-induced cardiac ischemia.

A 48-year-old woman with uterine cervical cancer with no cardiovascular risk factors was admitted to our hospital for concurrent chemoradiotherapy (CCRT) and planned to receive weekly paclitaxel and carboplatin for a total of 5 weeks. Just after the completion of the first cycle of paclitaxel infusion, she presented with diaphoresis and her consciousness level decreased. Electrocardiography showed ST elevation, suggesting acute myocardial infarction. Laboratory testing revealed troponin I positivity. Emergency coronary angiography (CAG) revealed a normal coronary artery, suggesting paclitaxel-induced vasospasm. After CAG, the patient was hemodynamically stable and was returned to the gynecologic unit two days after CAG. CCRT without paclitaxel was continued and the patient was uneventfully discharged from hospital.

## Introduction

Paclitaxel is one of the most important chemotherapeutic agents in gynecologic oncology. It acts by promoting cellular death by inhibiting the microtubule stabilization and interfering with polymerization dynamics, thereby inducing the arrest of mitosis. [1]. In addition to hypersensitivity reactions, toxicities encountered during paclitaxel treatment include neurological, hematological, gastrointestinal, and cardiac toxicities. Paclitaxel-induced cardiac ischemia is a rare but life-threatening complication. Although it may be difficult to distinguish between hypersensitivity reactions and infusion reactions, it should not be overlooked. A delay in the diagnosis of paclitaxel-induced cardiac ischemia can be fatal.

We herein report a rare case of ST-segment elevation following the administration of paclitaxel for uterine cervical cancer. We also present the clinical course of our case and review the relevant literature on paclitaxel-induced cardiac ischemia.

## Case presentation

A 48-year-old woman with uterine cervical cancer (adenocarcinoma, stage IIB) with no cardiovascular risk factors was admitted to our hospital for concurrent chemoradiotherapy (CCRT) consisting of weekly carboplatin plus paclitaxel, external beam radiotherapy (EBRT), and intracavitary brachytherapy (ICBT). The administration of weekly paclitaxel (35 mg/m2) and carboplatin (area under the blood concentration time curve [AUC] = 2) for a total of 5 weeks was planned, based on previous studies [2, 3]. EBRT targeting the whole pelvis at 2 Gy/fraction for 5 fractions/week, for a total of 25 fractions (50 Gy) was planned. The planned total dose of ICBT was 27.2 Gy in 4 fractions. The pretreatment serum CA19-9 level was 443 U/ml.

**Fig. 1:**
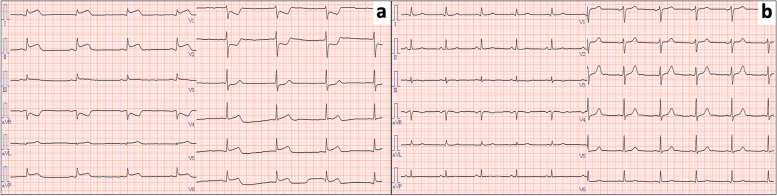
Electrocardiogram (ECG) after administration of paclitaxel. **a** ECG during coronary vasospasm. ST elevation in the leads I, II, III, aVL, aVF, V4, V5 and V6 ST elevation in the leads I, II, III, aVL, aVF, V4, V5 and V6. **b** ECG one day after coronary vasospasm. ECG reverted to normal

**Fig. 2:**
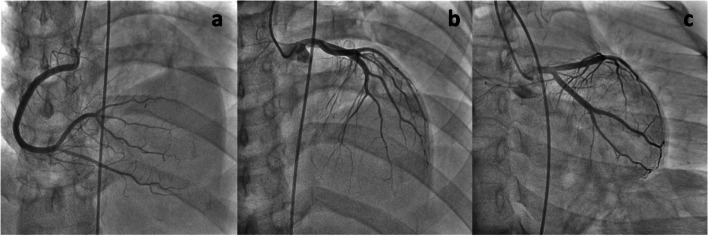
Emergency coronary angiography (CAG). CAG revealed normal coronary arteries. **a** Right coronary artery. **b** Left anterior descending coronary artery. **c** Left circumflex coronary artery

Just after the completion of the first cycle of paclitaxel infusion, the patient presented with diaphoresis and decreased consciousness level (Glasgow coma scale: 12). On examination, her heart rate was 40 beats/min, her blood pressure (BP) was 87/37 mmHg, and her oxygen saturation was 97% on room air. Prednisone and epinephrine were administered intravenously based on the assumption that the patient was exhibiting a hypersensitivity reaction. However, the patient did not recover and significant hypotension (systolic BP < 80 mmHg) continued. Electrocardiography (ECG) revealed ST depression in leads V1 and V2, and ST elevation in leads I, II, III, aVL, aVF, V4, V5 and V6, suggesting acute myocardial infarction (Fig. 1). Laboratory testing revealed troponin I positivity (0.097 ng/ml). The peak of Tropnin I was observed at four hours after disease onset (3.52 ng/ml). After the administration of dopamine for significant hypotension, she was transferred to the coronary care unit. Emergency coronary angiography (CAG) was performed and CAG revealed normal coronary arteries (Fig. 2), suggesting paclitaxel-induced vasospasm.

After CAG, the patient was hemodynamically stable and her ECG findings reverted to normal on the same day. She returned to the gynecologic unit two days after CAG. CCRT without paclitaxel was continued and the patient was discharged from hospital after the completion of CCRT. However, her serum CA19-9 level showed a marked increase to > 10,000 U/ml, and FDG-PET revealed multiple lymph node metastases and peritoneal carcinomatosis at one month after the completion of CCRT. Although irinotecan was administered as a second-line chemotherapy, the chemotherapeutic response was classified as progressive disease. Next-generation genome sequencing was performed using FoundationOne® CDx. (Foundation Medicine, Inc., Cambridge, MA, USA); however, we did not identify any targeted therapy options. Cancer-directed therapy was abandoned and the patient received palliative care. The patient died due to disease progression ten months after the diagnosis.

## Discussion

Acute side effects associated with paclitaxel, including allergic reaction, nausea, and cardiotoxicity, have been reported [4]. Hypersensitivity is a common adverse event associated with the administration of paclitaxel. Around 16–40% of patients develop a hypersensitivity reaction after receiving the injection [1]. In contrast, cardiotoxicity is considered to be a rare side effect in comparison to hypersensitivity or infusion reactions. Cardiac arrhythmias, including bradycardia or heart block (Mobitz type I and II, and complete heart block) are cardiotoxicities that are known to be related to the administration of paclitaxel [5]; the reported incidence is only 0.1%. Ischemic heart disease is an extremely rare but life-threatening adverse event that occurs in relation to the administration of paclitaxel.

We searched the PubMed database for all English-language articles related to paclitaxel-induced cardiac ischemia published by September 20, 2021 using the following key words and combinations of key words: “paclitaxel ST elevation” and “paclitaxel myocardial infarction”. Only 10 cases of paclitaxel-induced cardiac ischemia have been previously reported [6–15]. Of these 10 cases, we were able to obtain detailed information for 8 patients (Table 1). Most cases occurred within several hours after the administration of paclitaxel (patient nos. 1–4, 6–8) [8–11, 13–15] and it even occurred in patients without cardiovascular risk factors (patient nos. 2, 4, 6 and 7) [9, 11, 13, 14]. Although there is no universally accepted evidence regarding paclitaxel-induced vasospasm, vasodilator for example calcium blocker or nitroglycerin would be preferred when the patient is hemodynamically stable. One patient was initially treated with intravenous prednisone or epinephrin, under the assumption that the patient was exhibiting a hypersensitivity reaction (patient no. 2) [9]. It is difficult to distinguish ischemic heart disease from hypersensitivity or infusion reactions, because the clinical manifestations are very similar. Other authors experienced a case of recurrent paclitaxel-induced MI (patient no. 3) [10]. According to the previous report, the re-administration of paclitaxel for patients with a history of paclitaxel-induced MI seems to be unacceptable because of the risk of recurrent MI.

**Table 1:** Summary of patients with paclitaxel-induced cardiac ischemia

Case	Reported Year	Author	Cancer type	Age	Sex	Cardiovascular risk factor^a^	Cycle of chemotherapy	Symptom	Onset	ECG changes	Coronary angiography	Treatment	Short term outcome
1	1996	Hekmat	Breast cancer	67	Female	Previously smoked	Second cycle	Chest pain	Fifteen hours after paclitaxel infusion	ST elevation in leads II, III and aVF	Not performed	Medical management only (Nitroglycerin)	Dead
2	2005	Schrader	Ovarian cancer	58	Female	None	First cycle	Chest pain, nausea	20 min after paclitaxel infusion	ST elevation in leads II, III and avF	Not performed	Medical management only (Intraveneaous heparin)	Alive
3	2009	Gemici	Ovarian cancer	51	Female	Previous history of myocardiac infarction after paclitaxel administration	Second cycle	Chest pain, diaphoresis	Within minutes of paclitaxel administration	ST elevation in leads II, III, aVF, V3, V4, V5 and V6	80% stenosis of the left circumflex	Stent placement	Alive
4	2009	Londhey	Ovarian cancer	48	Female	None	Fifth cycle	Sudden circulatory collapse	Just after completion of paclitaxel infusion	ST elevation in lead V2	Not performed	Medical management only (Intraveneaous heparin, oral aspirin)	Alive (Discharged after a week)
5	2009	Park	Ovarian cancer	63	Female	Hypertension	First cycle	Chest pain	Next day after paclitaxel administration	ST elevation in leads V2, V3, V4 and V5	Filling defect in the left main coronary artery and 100% stenosis of the distal left anterior descending artery	Balloon angioplasty	Alive
6	2012	Shah	Ovarian cancer	45	Female	None	First cycle	Left-sided heaviness in the chest	3 h after the completion of paclitaxel infusion	ST depression in leads V1, V2 and aVL	Not performed	Medical management only	Alive (Discharged from hospital after 10 days)
7	2014	Esber	Breast cancer	47	Female	None	Second cycle	Facial flushing, chest pain	Within 5 min of paclitaxel administration	ST elevation in leads V1, V2 and V3	95% stenosis of the proximal left anterior descending artery	Drug-elutinting stents placement	Dead
8	2016	Rawal	Esophageal cancer	63	Male	NA	NA	Chest Pain, breathlessness and hypotension	Just after completion of paclitaxel infusion	ST elevation in leads II, III and aVF	100% stenosis of the proximal right coronary artery	Stent placement	Alive
Current case	2022	Higami	Cervical cancer	48	Female	None	First cycle	Fatigue, diaphoresis	Just after completion of paclitaxel infusion	ST elevation in leads I, II, III, aVL, aVF, V4, V5 and V6	Normal coronary angiography	Medical management only	Alive (but died of disease progression 10 months after diagnosis)

*NA* Not available

^a^Diabetes, hypertension, history of coronary heart disease, obesity or smoking

The exact mechanism that leads to myocardial ischemia in patients receiving paclitaxel is not clearly described in the literature. It has been claimed that paclitaxel may interfere with intracellular calcium regulation and alter cardiac activity [16]. Additionally, the conventional formulation containing Cremophor® may contribute to cardiac toxicity by inducing histamine production. [17]. Cremophor stimulates H1 and H2 receptors leading to increased myocardial oxygen demand and coronary vasoconstriction. According to another previous report, the Rho-kinase pathway and its activation are known to play a central role in the molecular mechanism of coronary artery vasospasm. [18]. Paclitaxel increases the Rho-kinase expression and its activity [19] and paclitaxel-induced coronary artery vasospasm may occur.

In conclusion, paclitaxel-induced cardiac ischemia is a rare but life-threatening complication that can occur in any patient treated with paclitaxel, even in the absence of cardiovascular risk factors. Physicians should recognize the importance of avoiding delays in the diagnosis and treatment.

## Data Availability

All of the material used during the manuscript are available from the corresponding author on reasonable request.
